# Cortical maturation in children with cochlear implants: Correlation between electrophysiological and behavioral measurement

**DOI:** 10.1371/journal.pone.0171177

**Published:** 2017-02-02

**Authors:** Liliane Aparecida Fagundes Silva, Maria Inês Vieira Couto, Fernanda C. L. Magliaro, Robinson Koji Tsuji, Ricardo Ferreira Bento, Ana Claudia Martinho de Carvalho, Carla Gentile Matas

**Affiliations:** 1 Department of Physical, Speech and Occupational, FMUSP, São Paulo (SP), Brazil; 2 Department of Otorhinolaryngology, Clinical Hospital, FMUSP, São Paulo (SP), Brazil; University of California Irvine, UNITED STATES

## Abstract

Central auditory pathway maturation in children depends on auditory sensory stimulation. The objective of the present study was to monitor the cortical maturation of children with cochlear implants using electrophysiological and auditory skills measurements. The study was longitudinal and consisted of 30 subjects, 15 (8 girls and 7 boys) of whom had a cochlear implant, with a mean age at activation time of 36.4 months (minimum, 17 months; maximum, 66 months), and 15 of whom were normal-hearing children who were matched based on gender and chronological age. The auditory and speech skills of the children with cochlear implants were evaluated using GASP, IT-MAIS and MUSS measures. Both groups underwent electrophysiological evaluation using long-latency auditory evoked potentials. Each child was evaluated at three and nine months after cochlear implant activation, with the same time interval adopted for the hearing children. The results showed improvements in auditory and speech skills as measured by IT-MAIS and MUSS. Similarly, the long-latency auditory evoked potential evaluation revealed a decrease in P1 component latency; however, the latency remained significantly longer than that of the hearing children, even after nine months of cochlear implant use. It was observed that a shorter P1 latency corresponded to more evident development of auditory skills. Regarding auditory behavior, it was observed that children who could master the auditory skill of discrimination showed better results in other evaluations, both behavioral and electrophysiological, than those who had mastered only the speech-detection skill. Therefore, cochlear implant auditory stimulation facilitated auditory pathway maturation, which decreased the latency of the P1 component and advanced the development of auditory and speech skills. The analysis of the long-latency auditory evoked potentials revealed that the P1 component was an important biomarker of auditory development during the rehabilitation process.

## Introduction

Hearing loss is an impairment that impedes full reception of the acoustic signal by the auditory cortex because it reduces stimulation of the auditory pathways. In childhood, severe or profound, hearing loss affects the development or maintenance of oral language and, consequently, the individual's relationships and lifestyle [1,2].

Cochlear implants (CI) are an important clinical resource for children with hearing loss who do not show significant results in auditory skill development with the use of only a hearing aid [3]. This electronic device is meant to partially replace the sensory function of the cochlea through direct electrical stimulation of the auditory nerve fibers, which allows access to speech sounds and thereby improves the quality of life of these patients [4,5].

Auditory stimulation during childhood allows the central auditory nervous system (CANS) to undergo changes and reorganization, called neuronal plasticity, that facilitate the development of auditory skills (detection, discrimination, recognition and comprehension) that are necessary for oral language development [6–13].

However, the behavioral auditory and language response after CI activation is not always immediate and precise because progress is gradual and results can be influenced by many variables, such as sensory deprivation time, age at activation, degree and type of hearing loss, acquisition time (pre- or post-lingual), etiology, the presence of residual hearing, speech therapy, family motivation and involvement, and the presence of other comorbidities that can affect the maturation process (syndromes or delays in general psychomotor development) [14].

Furthermore, optimization of the CI's benefit does not depend only on these variables or on the electrical signal generated by the device. The integrity of the central auditory pathways that carry sound information to the primary auditory cortex (located in Heschl’s gyrus) and the ability of other associated areas, such as the secondary auditory area (which extends to the lateral surface of the temporal lobe) and Wernicke's area (which includes part of the temporal plane and posterior superior part of the first temporal gyrus), can significantly affect optimization; both factors are related to auditory learning and allow the CI user to add meaning to acoustic signals [15,16].

The existence of a sensitive period for early stimulation aiming for a greater CI benefit is discussed in the literature, and in general, the consensus is that up to three years of age is the perfect time to start the process of (re)habilitation [17,18,19]. After this sensitive period, abnormalities in the development of synaptic plasticity occur, resulting in abnormal neuronal connectivity among cells, as well as disintegration and functional immaturity of auditory cortical areas [11,12,18]. Thus, the feedback between the primary and secondary auditory areas is impaired, and some auditory areas can develop non-auditory functions such as visual and somatosensory functions, a phenomenon called cross-modal plasticity [17,20–26].

To evaluate the development of auditory and language skills, specific protocols are used and should be selected according to the child's age and developmental level. Besides that, questionnaires should be given to parents or guardians to provide quantitative and qualitative feedback regarding the child's performance in daily life [27,28,29].

Considering the subjective nature of behavioral measures, evaluation by means of long-latency auditory evoked potentials (LLAEPs) has recently emerged as a way to objectively evaluate the benefits provided by the CI to complement behavioral evaluation. This test can measure the degree of development and plasticity limits of central auditory pathways by analyzing changes in the morphology and P1 component latencies present in this potential [11,18,26,30–35], generated by the electrical activity of the primary auditory cortex and associated thalamic regions [12]. Therefore, the evaluation of LLAEPs in conjunction with behavioral auditory and language measurements could be valid for monitoring the development of auditory and oral language skills in hearing-impaired children after the intervention and could therefore help to establish treatment guidelines [18,19,32,34,36–43].

However, studies that determine the correlation between behavioral auditory and language measurements and electrophysiological evaluations are still insufficient for LLAEP evaluations to be used in clinical practice, particularly in patients who are difficult to evaluate using behavioral measures, for reasons of age, restricted auditory experience or any other factor related to cognition and development. Despite several studies in recent decades that have aimed to characterize LLAEP findings in children with CI, the heterogeneity of this population makes it difficult to establish a consensus on what to expect from electrophysiological results after CI activation.

The objective of the present study was to monitor the cortical maturation of children with CI by means of electrophysiological and auditory and speech skills measurements.

## Methodology

### Study type and ethical aspects

This was a longitudinal clinical study conducted at the Department of Physical, Speech and Occupational Therapy in conjunction with the CI Group of the Clinical Hospital, School of Medicine, University of São Paulo (Hospital das Clínicas da Faculdade de Medicina da Universidade de São Paulo–HCFMUSP).

The study was approved by the Ethics Committee for Analyses of Research Projects (Comissão de Ética para Analises de Projetos e Pesquisas—CAPPesq), HCFMUSP under process number 0319/11, and the procedures were performed after the children's parents or guardians signed an informed consent form.

### Sample

The sample included 30 children of both genders, with a maximum age of 5 years and 11 months, who were divided equally into two subgroups as described below:

#### Children with CI

This group consisted of 15 children, 8 of whom were female and 7 were male, who ranged in age from 17 to 66 months (36.4 ± 9.51 months). Hearing aid usage prior to CI surgery was from 1 to 36 months (15.73 ± 13.79 months). The duration of deafness was from 6 to 42 months (19.73 ± 9.55 months). The CI companies included five children who were users of CI from Cochlear Corporation, four children were users of CI from Med-el, two children were users of CI from Neurelec, and three children were users of CI from Advanced Bionics. Regarding etiology, ten children had an unknown etiology, three had genetic changes, and two by ototoxicity drug effects. All children used hearing aids in the contralateral ear.

Sample selection of children with CI considered the following inclusion criteria: children up to five years of age with severe and/or profound bilateral prelingual sensorineural hearing loss who had undergone unilateral CI surgery and who had full insertion of electrodes, effective use of the CI (eight hours or more per day, according to information provided by the parents), regular attendance at speech therapy (once or twice a week), residence in the state of São Paulo, and absence of neurological or cognitive impairment or other changes that could affect auditory and language development.

#### Hearing children

This group consisted of 15 children who were matched with those children with CI according to chronological age and gender, with an age range from 17 to 63 months (36.9 ± 13.39 months). The criterion used for age pairing was an age difference of up to three months between the two children.

Contact was made with kindergarten directors, who were asked to allow an invitation letter to be sent to each student's guardian, and with friends and family who were willing to bring their children to participate.

The inclusion criteria were children with normal hearing, whose hearing loss was considered negligible according to the normalcy criteria of the following tests: acoustic impedance measurements with tympanometric curve type A with ipsilateral and contralateral acoustic reflexes at 500, 1000, 2000 and 4000 Hz [44]; a speech recognition threshold with words or simple commands at less than 20 dB HL [45]; and behavioral tests that were selected according to the degree of the child's understanding: conventional pure-tone audiometry or conditioned audiometry at frequencies of at least 500 to 4000 Hz, with a lower auditory threshold of 20 dB HL at all tested frequencies. With the youngest child, it was necessary to perform a brainstem auditory evoked potential test to complement the behavioral evaluation results, and the adopted normalcy criterion was the presence of waves I, III and V at 80 dBnHL for clicks with absolute latencies and interpeaks within the normal range for the age group and electrophysiological threshold for clicks at 20 dBnHL bilaterally.

In addition, children did not present cognitive, neurological, motor or language impairments and did not undergo prior speech therapy, which was assessed through interviews with the child's mother, as well as through the observation of a speech therapist.

### Procedures

After the children's medical records were analyzed, those who fit the inclusion criteria were invited to participate in the study through an invitation letter delivered to the child's guardian or through telephone contact.

Each child was evaluated at two time points: after three months of using the CI and after nine months of experienced CI use. The same time interval was followed for hearing children.

Only the children with CI were evaluated regarding the auditory and speech skills using instruments that are available in Brazilian Portuguese: the Infant-Toddler Meaningful Auditory Integration Scale (IT-MAIS/MAIS) [28,46], and the Meaningful Use of Speech Scales (MUSS) [27,47]. Both of the instruments consist of structured interviews, which are composed of 10 questions that are applied to parents. Each question should receive a score between zero and four points (0 = never; 1 = seldom; 2 = occasionally; 3 = frequently; 4 = always). The scores for each question were summed, with a maximum score of 40 points (converted into a percentage). The Glendonald Auditory Screening Procedure Protocol (GASP) [48,49], which was adapted to Brazilian Portuguese, was used to evaluate the speech perception of children and determine their listening skills. This protocol consists of six tests that evaluate variations from the detection to speech comprehension skills: Ling sound detection (test 1), discrimination of a male or female voice (test 2), vowel discrimination (test 3), discrimination of vowel extension (test 4), closed recognition of words (test 5), and comprehension of simple sentences (test 6). The six tests were applied successively, and in order to move to the next test, the child must have had an accuracy of 50% or more on the previous test. Detection skill was considered when test 1 was completed. Discrimination skill was contemplated when tests 2, 3 and 4 were completed. Recognition skill was considered when test 5 was fully completed. Finally, the comprehension skill was considered when test 6 was completed.

The LLAEP evaluation was performed on both groups in an acoustically treated room with the child in a state of alertness and sitting comfortably in a reclining chair. The child was guided and encouraged to watch a movie without sound during the procedure. Before beginning the procedure, the function of the CI was assessed.

The equipment used was the Smart EP USB Jr (IHS 5020; Intelligent Hearing Systems). This system uses two stimulation channels, channel A for right-sided capture and channel B for left-sided capture.

After cleaning the child's skin with electroencephalogram (EEG) abrasive cleaning gel, electrodes were attached with EEG conductive paste. The electrode placements followed standard IES 10–20 guidelines from the International Electrode System [50]. The accepted electrode impedance level for the procedure was between 1 and 3 kΩ.

Acoustic stimulation was presented in a sound field system with boxes positioned at an azimuth of 90° that was 40 cm away from the CI child’s implanted side. The same procedure was performed with the hearing children, and the stimulus was presented on the side with better hearing thresholds.

Regarding the stimulation parameters, the LLAEPs were recorded by means of /ba/ synthesized speech syllable stimulus with a total duration of 114.88 ms (75 ms of vowel duration and 18 ms consonant duration), an initial pitch of 112.4 Hz and final pitch of 111.2 Hz, which was composed of the following formants: F1 = 818 Hz; F2 = 1,378 Hz; F3 = 2,024 Hz; F4 = 2,800 Hz; F5 = 4,436 Hz.

A total of 512 stimuli were presented in each scan at an intensity of 70 dBnHL with a presentation rate of 1.9 stimuli per second and an inter-stimulus interval of 416 ms with alternating polarity. Other parameters were also used during recording: a 1–30 Hz bandpass filter, 100,000 gain, and a 0-ms pre-stimulus and 500-ms post-stimulus response analysis window.

Two traces were collected from each subject to confirm the results. Each trace was evaluated by two judges (audiologists with clinical experience in LLAEP analysis), who individually carried out blinded analyses with no identification of subjects. A third judge was consulted in cases where there were divergences on the initial analysis.

The criterion for identification and analysis of the P1 component was based on the literature [18] and considered the first and robust positive peak (largest amplitude) that had latency values between 50 and 300 ms and that presented reproducibility [37].

The study variables for the auditory and speech skills evaluated in the children with CI were the percentages obtained in the two questionnaires (IT-MAIS/MAIS and MUSS) as well as the classification of auditory skills obtained by performing the GASP (detection, discrimination, recognition and comprehension).

With regard to the electrophysiological auditory evaluation in both groups, the results obtained for the P1 component latencies present in the LLAEPs were analyzed throughout the six-month follow-up period in both groups.

The data collected were statistically analyzed using SPSS version 22. Descriptive analyses were performed using the mean, minimum, maximum, median and the 1st and 3rd quartiles. Because the data were not normally distributed, comparisons were performed using non-parametric tests. An inter-group comparison was performed using the Mann-Whitney test. Spearman's correlation coefficient was used to investigate the relationship between the measurements. The significance level adopted was 5%, and p-values considered statistically significant were marked with an asterisk (*).

## Results

### Auditory and speech skills in children with CI

**Fig 1:**
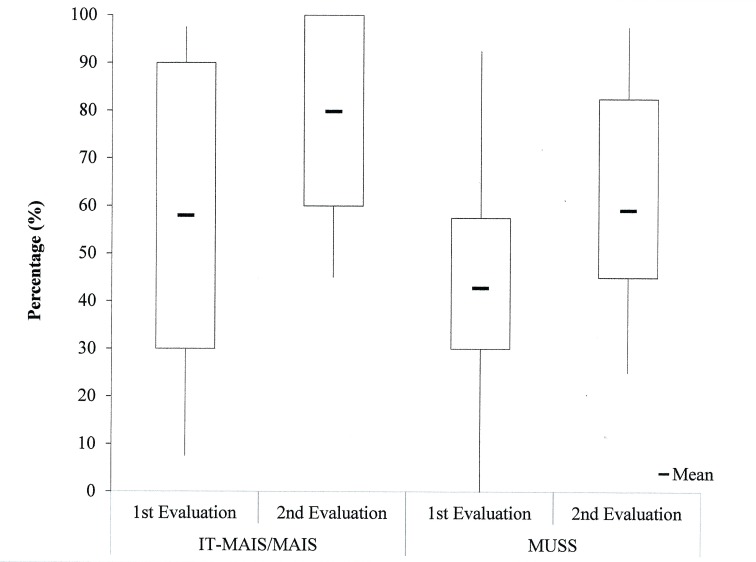
Descriptive analysis of the percentages obtained in the IT-MAIS/MAIS and MUSS questionnaires for the children with CI at both time points.

The results of auditory and speech skills from the perspective of the parents (IT-MAIS/MAIS and MUSS questionnaires) at both time points indicated progress in the course of auditory stimulation via CI in all evaluated cases (Fig 1). The mean values of the IT-MAIS/MAIS were 58.0% at the 1st evaluation and 79.8% at the 2nd evaluation. For the MUSS questionnaire, the mean values were 42.8% at the 1st evaluation and 59.2% at the 2nd evaluation.

There was a difference in the IT-MAIS/MAIS questionnaire between the first and second evaluation (Z = -3.410; p = 0.001), where the highest median was observed at the 2nd evaluation. Likewise, for the MUSS, there was a difference between the 1st and the 2nd evaluation (Z = -3.357; p = 0.001), where the score for the first evaluation was significantly lower, which indicates that the longer the activation time, the better the behavioral outcomes from the parents' perspective.

There was a positive correlation between the IT-MAIS/MAIS and MUSS protocols at both time points, which indicates that the increase in scores in the IT-MAIS/MAIS protocol was related to the increase in the MUSS score (Table 1).

**Table 1:** Correlation between the IT-MAIS/MAIS and MUSS questionnaires in the children with CI at both time points.

		IT-MAIS/MAIS	
		1^st^ evaluation	2^nd^ evaluation
**MUSS**	**1**^**st**^ **evaluation**	ρ = 0.871	ρ = 0.740
		p<0.001*	p = 0.002*
	**2**^**nd**^ **evaluation**	ρ = 0.927	ρ = 0.715
		p = <0.001*	p = 0.003*

* significant difference: p≤0.05, Spearman's correlation coefficient.

In relation to the behavioral data, the GASP performance revealed that the auditory skills achieved after nine months of CI use were detection (53.5% of cases) followed by discrimination (40% of cases); comprehension skills were observed in only one case (6.7%).

### Electrophysiological auditory evaluation

**Fig 2:**
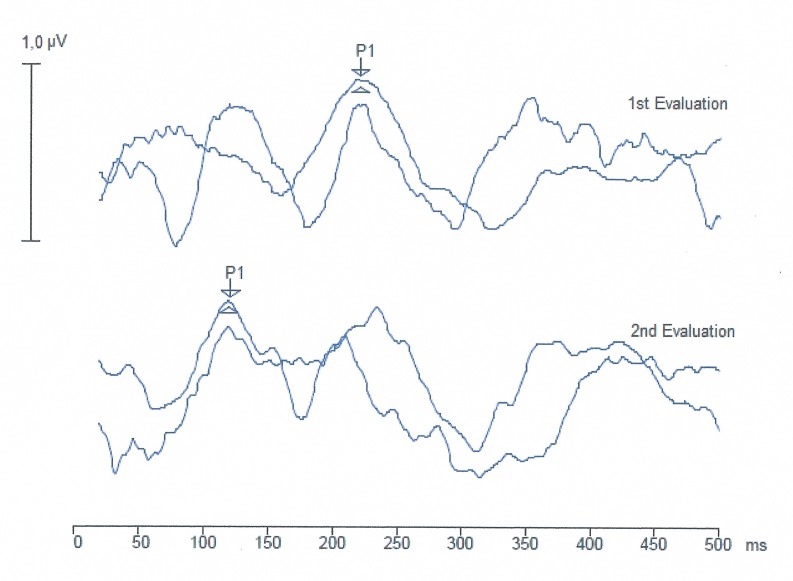
LLAEP trace recording of a child using CI at the two time points. ms, milliseconds; μV, microvolts.

The LLAEP trace analysis revealed the presence of the P1 component in 100% of the children in both groups. In the children with CI, the trace became more defined after nine months of auditory experience with CI use (Fig 2).

**Fig 3:**
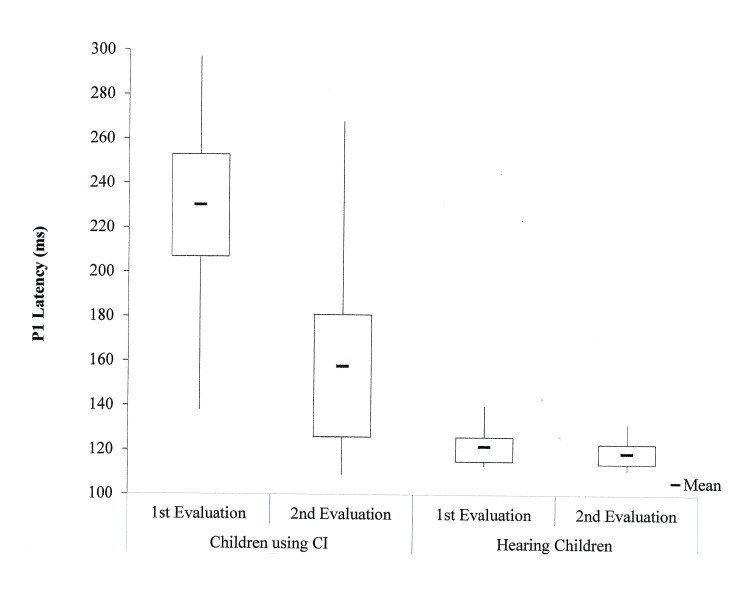
Descriptive statistics of P1 component latencies (ms) at the two time points for both groups. ms, milliseconds; CI, cochlear implants.

The results of the LLAEP analysis in both groups showed that over time, there was a decrease in P1 component latency values in both groups (Fig 3). Mean values of the P1 component latency in children with CI was 230.3 ms in the 1st evaluation and 157.9 ms in the 2nd evaluation. For hearing children, the mean values of the P1 component latency were 121.9 ms in the 1st evaluation and 118.9 ms in the 2nd evaluation.

Comparison of the electrophysiological evaluation between the two time points revealed a significant difference both in children with CI (Z = -3.408; p = 0.001) and in hearing children (Z = -2.869; p = 0.004). No between-group interactions were observed in the latency values over time.

The reduction in latency between evaluations was more evident in the children with CI, which suggests that after nine months of auditory experience, there was a significant change in latency values.

To compare the latency values obtained for each group and at each time point, the Mann-Whitney test was used. There was a significant difference in P1 component latency values between both groups in the 1st (U = 2.0; Z = -4.587; p < 0.001) and the 2nd (U = 36.0; Z = -3.155; p = 0.001) evaluations, which demonstrates that nine months of CI use was still insufficient to achieve equivalent latency values between the groups.

### Comparison of auditory, speech and electrophysiological measures in children with CI

The Spearman's correlation coefficient was used to compare LLAEP P1 component latencies with the responses of auditory and speech skills in the children with CI at both time points. The correlation analysis between P1 component latency and auditory skills using the IT-MAIS/MAIS questionnaire indicated statistical significance only for P1 latency in the second evaluation using the IT-MAIS/MAIS questionnaire score in the 1st and 2nd evaluations. In both cases, the correlation was negative, which suggests that a reduction in P1 latency occurred when the IT-MAIS/MAIS score increased.

**Fig 4:**
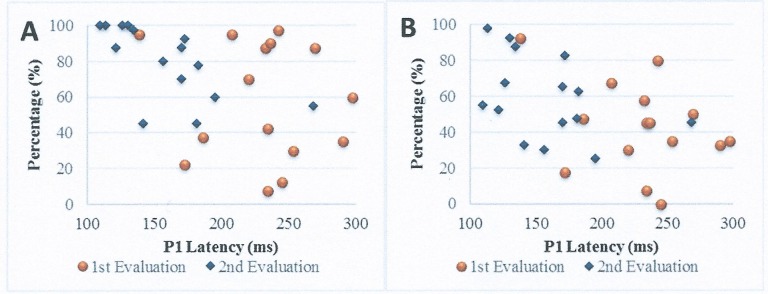
Scatter plots showing the longitudinal correlation between the electrophysiological measurement and IT-MAIS (A) and between the electrophysiological measurement and MUSS (B). ms, milliseconds.

Regarding the MUSS questionnaire, there was no significant correlation with the electrophysiological evaluation at either time point (Table 2; Fig 4).

**Table 2:** Correlation between P1 component latencies and IT-MAIS/MAIS and MUSS scores in the children with CI at both time points.

	IT-MAIS/MAIS		MUSS	
	1^st^	2^nd^	1^st^	2^nd^
	evaluation	evaluation	evaluation	evaluation
**P1 latency**	ρ = -0.165	ρ = -0.190	ρ = -0.252	ρ = -0.106
**1**^**st**^ **evaluation**	p = 0.557	p = 0.498	p = 0.366	p = 0.708
**P1 latency**	ρ = -0.654	ρ = -0.740	ρ = -0.388	ρ = -0.471
**2**^**nd**^ **evaluation**	p = 0.008*	p = 0.002*	p = 0.153	p = 0.076

* significant difference: p≤0.05, Spearman's correlation coefficient.

**Fig 5:**
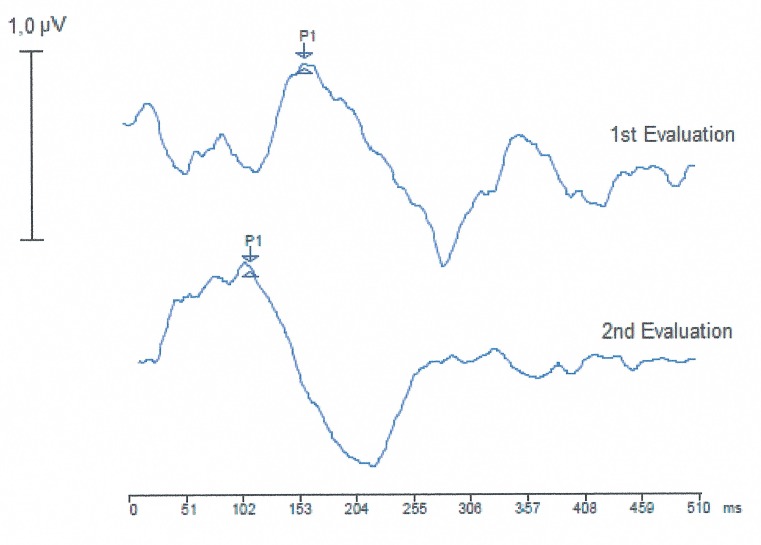
LLAEP trace recording of a child with CI with comprehension skills at the two time points. ms, milliseconds; μV, microvolts.

Because the auditory skills obtained on the GASP consisted predominantly of detection and discrimination, it was impossible to investigate the correlation. However, it was decided to subdivide the children with CI according to the auditory skills achieved (detection or discrimination) and to compare these subgroups with the results obtained in the remaining evaluations using Mann-Whitney tests. Because only one child had developed the skill of comprehension, it was impossible to include her in this statistical analysis. It is noteworthy that this child also showed better results in the electrophysiological evaluation, with lower latency values since the first evaluation (Fig 5).

Performance on the three measures (P1 component latencies and IT-MAIS/MAIS and MUSS questionnaire scores) in the second evaluation was convergent with the classified auditory skills. The subgroup that achieved the better auditory skill (discrimination) had a lower P1 latency value and higher IT-MAIS/MAIS and MUSS scores (Table 3).

**Table 3:** Comparison of performance in the second evaluation according to auditory skills obtained in the GASP in the children with CI.

Measure	Auditory skill	Median	1st	3rd	U	z	p
	(GASP)		quartile	quartile			
**P1 latency**	Detection	175.50	159.50	191.75	4.0	-2.585	0.010*
	Discrimination	128.00	118.00	143.50			
**IT-MAIS/ MAIS**	Detection	65.00	47.50	79.38	0.5	-3.054	0.002*
	Discrimination	98.75	91.25	100.00			
**MUSS**	Detection	45.00	30.63	58.75	4.0	-2.585	0.010*
	Discrimination	75.00	54.38	88.75			

* significant difference: p≤0.05, Mann-Whitney test.

## Discussion

The overall objective of this study was to monitor the cortical maturation of children with CI by means of electrophysiological and auditory and speech measurements.

This longitudinal study evaluated a significant number of cases, a small age range, and a carefully defined control group that was assessed by the same methodology in order to ensure the best possible comparison.

The analysis and comparison of the electrophysiological and behavioral measures provide a broad view regarding how much the electrophysiological assessment could predict behavioral changes due to auditory stimulation through CI.

### Auditory and speech skills in children with CI

It is known that the development of auditory skills occurs gradually and is dependent on central auditory pathway maturation. Through the process of auditory (re)habilitation (via CI and speech therapy), access to speech sounds provided by the CI enables new neuronal connections to be established in the CANS. These connections, in turn, are increasingly strengthened, which gradually promotes the development of auditory skills [14].

After nine months of monitoring auditory development in children with CI, parents were able to perceive gradual changes in auditory skills as measured by the IT-MAIS/MAIS questionnaire.

In general, the mean scores in the IT-MAIS/MAIS in both evaluations, in general, corroborate the findings of previous studies [51–53]. According to the literature, it is expected that children will achieve an IT-MAIS percentage of approximately 30% after three months of auditory experience with the use of the CI. After 10 months, this percentage can increase to approximately 70% [54].

These data suggest that according to the parents' perspective, as auditory experience increases with CI use, a concomitant development in auditory skills can be observed. After nine months of CI use, the GASP results showed that 53.3% of children achieved the auditory skill of detection, 40% achieved the skill of discrimination, and only one child (representing 6.7%) achieved the skill of comprehending speech.

For the development of more complex auditory skills, longer experience with the CI is generally required. It is known that the development of auditory skills goes beyond access to sounds; despite the CANS being highly plastic, central auditory pathway maturation is dependent on intrinsic factors, which are related to individual susceptibility and extrinsic factors that relate to individual exposure to environmental variables [6]. In children with CI, there are many intrinsic and extrinsic factors that clearly affect the results of the (re)habilitation, such as sensory deprivation time, the age at implantation, speech therapy, effective auditory stimulation, motivation and family commitment, cognitive functions of attention and memory, learning ability, exposure to oral language prior to hearing loss, the presence of other alterations associated with hearing impairment, and the presence of residual hearing [14,55–57].

Another aspect evaluated in this study relates to speech skills from the perspective of the parents. It was noted that the benefits of CI were not restricted only to the auditory aspects, and due to the MUSS questionnaire, parents also reported an improvement in Other studies have observed scores between 7.5% and 17.5% before CI activation, 20% and 45% after three months of CI use, 35% and 50% after six months, and 62.5% and 70% after 12 months of use [51–53].

A positive correlation was observed in this study between the IT-MAIS/MAIS and MUSS questionnaires, which suggests a direct relationship between the development of auditory and speech skills. Taking this finding into consideration, it is possible that access to speech sounds provided by the CI enabled better auditory feedback, thereby increasing the children’s interest in oral language. The better access to sounds provided by the CI, combined with early diagnosis and intervention, favor the development of speech skills as incidental learning [10,58–61].

It is known that the heterogeneity of the population of children with CI strongly affects results [56]. Despite this diversity of characteristics and the variability in possible outcomes, increased development over time is always noticeable.

### Electrophysiological auditory evaluation

Analysis of the LLAEP evaluation data revealed that at the first evaluation, the P1 component was present in all children. LLAEP monitoring revealed noticeable CANS maturation after nine months of auditory experience via CI, in terms of better trace definition and the decrease in P1 component latencies observed in all children.

It is known that with sensory auditory stimulation, there are morphological and functional changes, such as an increase in the number of neurons that become responsive to sound stimuli, expanded dendritic branching, increased neuronal myelination and improved synaptic connections and synchronization [21,23,62]. These anatomical and physiological changes result in changes in the latency and morphology of LLAEP traces [21,23,62].

Although P1 component latency values also differ greatly in the literature [63], the data obtained in this study in both evaluations corroborate several findings reported by a study that evaluated P1 component latency in a group of children with a maximum age of 3 years and 5 months who received a CI and observed mean values of 378.18 ms at the time of activation and 137.5 ms after 12–18 months of CI use [19]. Another study monitored 10 children aged between one and five years and observed latency values of 313 ms in the pre-activation period and 259 and 177 ms after three and six months of CI use, respectively [64].

Considering the divergence of data in the literature regarding the normal pattern of LLAEP latency values in hearing children for the age group evaluated in this study (one to five years), as well as the diversity of protocols used to record this potential [65], it was decided to use the values obtained from the hearing children who were evaluated under the same conditions as the children with CI as a reference. It was observed that after a nine-month follow-up, the hearing children exhibited a significant decrease in P1 component latencies; however, when compared to the rate of decrease between the two time points across the two groups, there was clearly a greater decrease among children with CI.

A preliminary study that assessed five children before activation and after three months of CI use observed that this period of three months of CI use was also able to show differences in the latency values of the P1 component [66].

The CANS changes occurring as a result of CI electrical stimulation occur rapidly [18]. Several studies have observed children who approached the expected level for their age group three, four, six and eight months after the start of the CI interventional process [11,19,26,30,34]. In the present study, seven children with CI approached many of the values obtained in hearing children after nine months of stimulation. For the remaining children, P1 component latency values decreased over time but remained higher than those of the hearing children.

Because the rehabilitation process can directly influence the pace of development, the data obtained in this study suggest that the monitoring of children with CI should occur over the long term because for half of the children studied, nine months was not long enough to match the latency values obtained in hearing children.

Although there are many studies in this area, there is no consensus regarding what to expect from hearing children in this age group because of individual variables and the diversity of LLAEP recording protocols [65]. Moreover, in children with CI, for whom a greater number of variables can influence the results, the difficulty in standardizing latency values according to rehabilitation time is even greater.

The LLAEP analysis, which is primarily used for the longitudinal monitoring of central auditory pathway maturation, therefore seems to be an important clinical tool that can reflect the changes and plasticity of the CANS after therapeutic intervention. For this reason, many researchers have referenced P1 component analysis as a biomarker of post-intervention neurophysiological changes [30,43,62].

### Comparison of auditory, speech and electrophysiological measures in children with CI

Considering the diversity of results obtained in children with CI, different protocols must be used to better evaluate the results of the CI intervention. In recent decades, the literature has discussed the combined use of behavioral and electrophysiological measures to verify benefits and guiding treatment; however, little is known regarding the correlation between them. This is the first study described in the literature that compares data for the same electrophysiological assessment with three behavioral response measures.

In our study, when comparing the electrophysiological evaluation with the questionnaires given to parents, a correlation was found between the electrophysiological evaluation and the responses related to auditory skills from the perspective of the parents in the IT-MAIS/MAIS questionnaire. This finding corroborates other previously published studies [42,67]. It was observed that as P1 component latency decreased as auditory stimulation progressed, the IT-MAIS/MAIS score increased, which suggests that as auditory skills developed, a decrease in latency could be observed. Because the P1 component, which is present in the LLAEPs, is a response generated from bioelectric activity in the primary auditory cortex [55], its direct relationship to the development of auditory skills could be justified.

However, when longitudinally evaluating LLAEPs and the IT-MAIS protocol, other studies have observed decreased P1 component latency and increased IT-MAIS scores but with no significant correlation between the two tests. This finding emphasizes the need for more studies that compare the various protocols to better understand the results obtained by electrophysiological evaluation and behavioral response [64].

With respect to the responses of speech skills from the parents’ perspective, there was no significant correlation with LLAEPs as obtained by the MUSS questionnaire. Nevertheless, it is important to note that an increased MUSS score was observed alongside the reduction in P1 component latencies. As auditory development precedes the development of speech, a larger sample and/or a longer monitoring time could possibly identify other nuances that could not be observed in our sample within the nine months of follow-up.

Several studies have indicated that a reduction in P1 latency was correlated with improvements in communicative behaviors (vocalizations) [11], as well as auditory and language [26,37,43] In another study, evidence of improvement in LLAEPs in the first months of CI use indicated the presence of plasticity in the auditory system, which may precede an improvement in communication skills; children who exhibited progress in electrophysiological responses exhibited better speech comprehension and, therefore, greater success in auditory rehabilitation [8].

Regarding the auditory skills obtained by GASP evaluation, there was correlation between these and the other evaluated measures: children who achieved better auditory skill (discrimination) also had significantly lower P1 component latencies and higher IT-MAIS/MAIS and MUSS questionnaire scores than those who achieved only the detection skill. These findings reflect consistency in the results and suggest that speech perception was correlated with the parents' perception, as well as with the electrophysiological evaluation results.

It is known that the pace of development is not the same for all children and that results appear gradually over the course of the auditory experience [5]. Other studies following children over a longer period in terms of the CI auditory experience observed the gradual learning of other skills. One study showed that after a year of the CI auditory experience, most children were able to discriminate sounds of speech, and after six years, most were able to understand conversations without lip reading [68]. In a second study, it was found that in children who received their CI before the age of two, a gradual improvement in the GASP test was observed over time, but speech comprehension was only observed 18 to 24 months after the onset of electrical stimulation [69].

In the present study, only one child achieved the auditory skill of comprehension after nine months of CI use. The same child achieved latency values within those observed in hearing children of the same age. A single analysis of this subject suggests that a faster CANS maturation was able to facilitate this child's auditory and speech development more than the other children with CI.

Re-examination of the studies described in the literature revealed that a small number of subjects was a general limitation for many studies that analyzed LLAEPs in children with CI [8,11,37,43,64]. The reason for this limitation is that in addition to the difficulty in performing this procedure in children of this age group, an attempt must be made to form a somewhat homogeneous group, which thereby leads to a reduced number of sample subjects. Studies with larger numbers of subjects would therefore be important to confirm the findings presented here.

## Conclusion

The CI auditory stimulation enabled the gradual maturation of the CANS, which could be observed through changes in the LLAEP traces (decrease in P1 component latency values) as well as improve improvement in auditory and language speech skills from the parents’ perspective. (the development of auditory skills and oral language).

The LLAEP evaluation correlated with to the development of auditory skills and was found to be an important biomarker of CANS plasticity and functionality and a useful clinical tool for monitoring the benefits in of the rehabilitation process.

## Supporting information

S1 Table General information of all evaluations of all children. ms, milliseconds.
(PDF)

S1 Fig LLAEP record of all evaluations of all children. (TIF)
