# Correction: Cortical maturation in children with cochlear implants: Correlation between electrophysiological and behavioral measurement

**DOI:** 10.1371/journal.pone.0178341

**Published:** 2017-06-27

**Authors:** Liliane Aparecida Fagundes Silva, Maria Inês Vieira Couto, Fernanda C. L. Magliaro, Robinson Koji Tsuji, Ricardo Ferreira Bento, Ana Claudia Martinho de Carvalho, Carla Gentile Matas

There are errors in the author affiliations. The affiliations should appear as shown here: Liliane Aparecida Fagundes Silva^1*^, Maria Inês Vieira Couto^1^, Fernanda C. L. Magliaro^1^, Robinson Koji Tsuji^2^, Ricardo Ferreira Bento^2^, Ana Claudia Martinho de Carvalho^1^, Carla Gentile Matas^1^

**1** Department of Physiotherapy, Communication Science & Disorders, Occupational Therapy. Faculty of Medicine- University of São Paulo–FMUSP, Brazil, **2** Department of Otorhinolaryngology, Clinical Hospital—Faculty of Medicine University of São Paulo. FMUSP, Brazil.
