# Supplementary material for: Cortical maturation in children with cochlear implants: Correlation between electrophysiological and behavioral measurement
Source: PLoS One. 2017 Feb 2;12(2):e0171177. doi: 10.1371/journal.pone.0171177 (PMC5289550; doi:10.1371/journal.pone.0171177)
Supplement: S1 Table — ms, milliseconds. (PDF) [file pone.0171177.s001.pdf]

Subject	Hearing Children		Children with CI						
	P1 latency (ms)		P1 latency (ms)		IT-MAIS		MUSS		Auditory Skills
	1st evaluation	2nd evaluation	1st evaluation	2nd evaluation	1st evaluation	2nd evaluation	1st evaluation	2nd evaluation	2nd evaluation
**1**	114	114	234	195	7,5	60	7,5	25	Detection
**2**	139	129	220	121	70	87,5	30	52,5	Discrimination
**3**	132	132	172	141	22	45	17,5	32,5	Detection
**4**	140	124	186	170	37,5	87,5	47,5	45	Detection
**5**	126	121	290	268	35	55	32,5	45	Detection
**6**	117	116	232	172	87,5	92,5	57,5	82,5	Discrimination
**7**	118	111	269	126	87,5	100	50	67,5	Discrimination
**8**	113	113	138	113	95	100	92,5	97,5	Comprehension
**9**	123	122	253	181	30	45	35	47,5	Detection
**10**	118	118	297	170	60	70	35	65	Detection
**11**	117	116	245	156	12,5	80	0	30	Detection
**12**	118	118	242	130	97,5	100	80	92,5	Discrimination
**13**	124	123	207	134	95	97,5	67,5	87,5	Discrimination
**14**	114	113	234	182	42,5	77,5	45	62,5	Detection
**15**	115	114	236	109	90	100	45	55	Discrimination
