# Supplementary figures and images for: Cortical maturation in children with cochlear implants: Correlation between electrophysiological and behavioral measurement
Source: PLoS One. 2017 Feb 2;12(2):e0171177. doi: 10.1371/journal.pone.0171177 (PMC5289550; doi:10.1371/journal.pone.0171177)

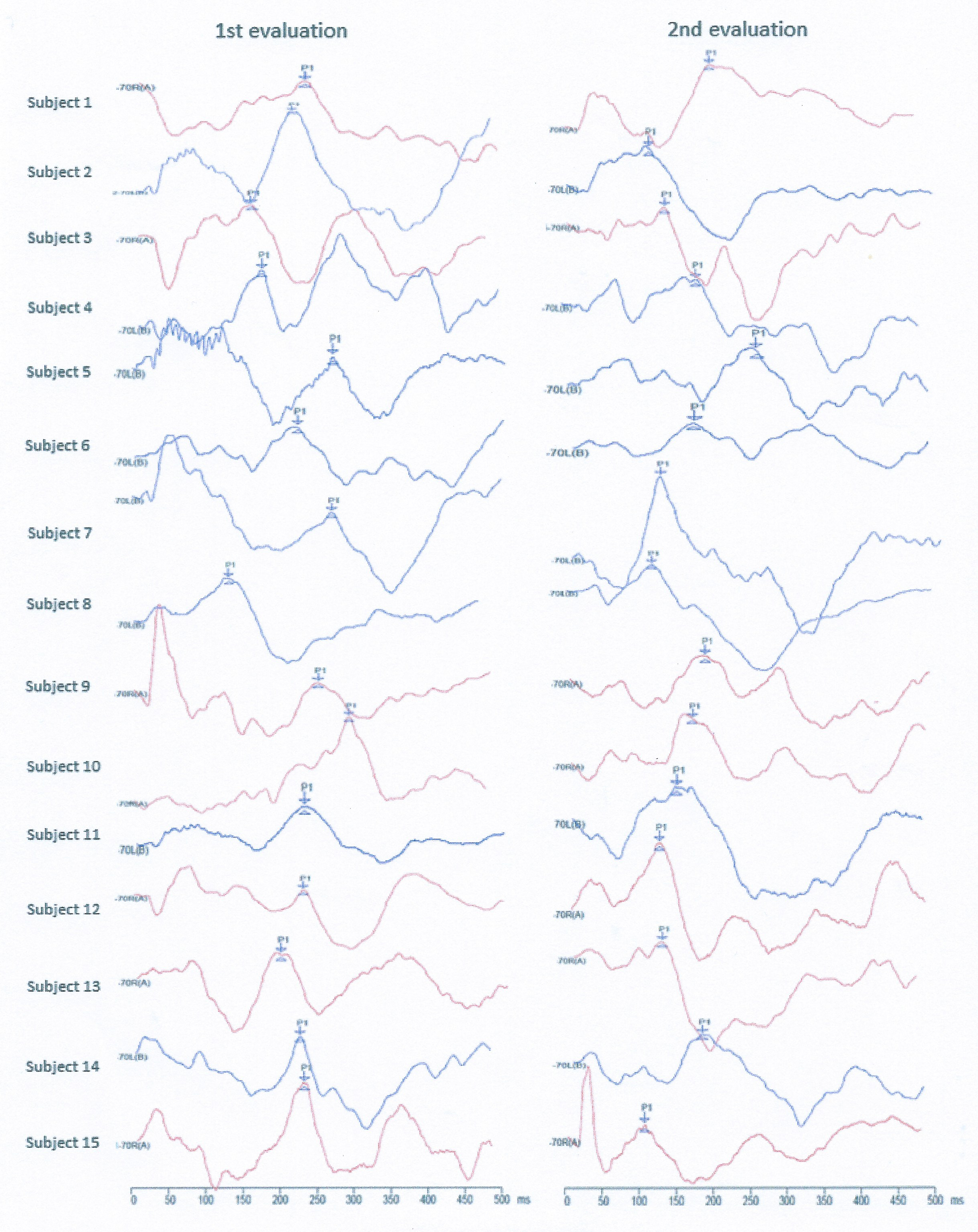

Supplement: S1 Fig — (TIF) [file pone.0171177.s002.tif]
